# The B subunit of *Escherichia coli* heat-labile toxin alters the development and antigen-presenting capacity of dendritic cells

**DOI:** 10.1111/jcmm.12599

**Published:** 2015-07-01

**Authors:** Jing Ji, Kristin L Griffiths, Peter J Milburn, Timothy R Hirst, Helen C O’Neill

**Affiliations:** a Research School of Biology, Australian National UniversityCanberra, ACT, Australia; b John Curtin School of Medical Research, Australian National UniversityCanberra, ACT, Australia; c Faculty of Health Sciences and Medicine, Bond UniversityGold Coast, QLD 4229, Australia

**Keywords:** dendritic cells, T cells, cell activation, adjuvant, enterotoxin

## Abstract

*Escherichia coli*’s heat-labile enterotoxin (Etx) and its non-toxic B subunit (EtxB) have been characterized as adjuvants capable of enhancing T cell responses to co-administered antigen. Here, we investigate the direct effect of intravenously administered EtxB on the size of the dendritic and myeloid cell populations in spleen. EtxB treatment appears to enhance the development and turnover of dendritic and myeloid cells from precursors within the spleen. EtxB treatment also gives a dendritic cell (DC) population with higher viability and lower activation status based on the reduced expression of MHC-II, CD80 and CD86. In this respect, the *in vivo* effect of EtxB differs from that of the highly inflammatory mediator lipopolysaccharide. In *in vitro* bone marrow cultures, EtxB treatment was also found to enhance the development of DC from precursors dependent on Flt3L. In terms of the *in vivo* effect of EtxB on CD4 and CD8 T cell responses in mice, the interaction of EtxB directly with DC was demonstrated following conditional depletion of CD11c^+^ DC. In summary, all results are consistent with EtxB displaying adjuvant ability by enhancing the turnover of DC in spleen, leading to newly mature myeloid and DC in spleen, thereby increasing DC capacity to perform as antigen-presenting cells on encounter with T cells.

## Introduction

Dendritic cells (DC) are antigen-presenting cells which take up foreign pathogens and present antigens to T cells, so initiating an immune response to the invading pathogen. In the context of vaccination, with the aim of pre-exposing the immune system to antigens from pathogens of interest, the type and magnitude of a T cell response can be modulated through use of adjuvants. Most adjuvants function at the time of antigen encounter with DC. They act either to stabilize the antigen and increase the probability of uptake by DC or to directly affect the DC, ensuring the antigen is encountered in an environment supporting a strong antigen-specific response.

Heat-labile enterotoxin (Etx), produced by enterotoxigenic *Escherichia coli*, and its non-toxic B subunit (EtxB) are known to be strong mucosal adjuvants although the mechanism by which they act as an adjuvant is not yet known 1. Enterotoxin is a hetero-oligomeric AB_5_ toxin with the non-toxic B subunit binding to the ganglioside GM1 on the surface of the cell facilitating translocation of the A subunit into the cytoplasm. Binding of EtxB to the cell surface appears to be an immunomodulatory process in itself 2 and GM1-binding by EtxB can up-regulate antigen presentation 3. While EtxB binds most strongly to GM1, it also binds to other cell surface molecules including asialo-GM1, GD1b-ganglioside and lactosylceramide. The contribution of these binding interactions to adjuvanticity is, however, not known 4. GM1 is ubiquitously expressed on most cells although expression varies across different cell types. For example, two subsets of monocytes with differing endocytic capacity can be identified based on the GM1 expression 5. Following EtxB binding, EtxA delivers a toxic effect into the cell cytoplasm through activation of the cyclic AMP (cAMP) pathway. In the epithelial cells of the intestine, this leads to the opening of ion channels and resulting diarrhoea 6.

Several previous studies have demonstrated the immunomodulatory effects of both Etx and EtxB, with emphasis on their adjuvanting properties 7–10. Previous studies on EtxB as an immunomodulator and adjuvant have focused on its role in T cell responses 11–13, and on its interaction with antigen-presenting cells like macrophages 14,15, and B cells 16,17. Previous studies on the interaction of EtxB with DC showed that binding to GM1 was essential for EtxB-mediated antigen presentation by JAWSII, an immortalized murine bone marrow-derived DC line 18. When DC generated in GM-CSF-induced bone marrow cultures were exposed to EtxB ovalbumin (-OVA) conjugates, they induced superior CD8^+^ T cell responses over OVA alone, but did not induce DC maturation 19. Despite these earlier studies, there has been no investigation of the effect of EtxB on DC *in vivo*.

A range of DC subsets can now be identified on the basis of their tissue of residence, functional capacity and marker expression. Splenic dendritic and myeloid subsets are very well characterized and can be identified on the basis of expression of the integrins CD11b and CD11c, as well as CD8α (or CD8) and MHC-II. Conventional (c)DC are CD11c^+^ MHC-II^+^ cells, with CD8^−^ CD11b^+^ and CD8^+^ CD11b^−^ subsets distinguishable. In terms of function, CD8^+^ cDC are cross-presenting, interleukin (IL)-12-producing cells resident in the T cell zone of spleen 20, while CD8^−^ cDC are located in the marginal zone, migrating to T cell zones following antigen encounter 21. Plasmacytoid (p)DC are circulating type I interferon-producing cells and can be identified by their CD11c^lo^ CD11b^−^ CD8^−^ MHC-II^+^ phenotype 22.

Here, we investigate the effect of EtxB administration on subsets of cDC and pDC and their precursors, as well as L-DC, myeloid cells and myeloid precursors *in vivo*. Furthermore, we investigate the ability of EtxB to enhance T cell activation upon administration to mice, and determine the importance of DC in EtxB-induced T cell activation *in vivo*.

## Materials and methods

### Animals

Specific pathogen-free C57BL/6J (*H-2K*^*b*^) female mice aged 6 weeks were obtained from the Australian National University (ANU) Biosciences Services (Canberra, ACT, Australia). BALB/c CD11c-diptheria toxin receptor (DTR) transgenic (tg) mice, C57BL/6.Tg(TcraTcrb)1100Mjb (OT-I) and C57BL/6.SJL/J.OT-II.CD45.1 (OT-II) were obtained from the Walter and Eliza Hall Institute (WEHI; Parkville, VIC, Australia). Mice were housed and handled according to protocols approved by the Animal Experimentation Ethics Committee at the ANU (Canberra, ACT, Australia). For tail vein injections (*i.v*.), substances were administered in 200 μl, using a 26G syringe. For intranasal vaccination, mice were anaesthetized by inhalation with 1-ml vaporized methoxyflurane (Penthrane™; Abbott Laboratories, North Ryde, NSW, Australia) and given 10 μl per nostril using a micropipette.

### Expression and purification of EtxB

Cultures of *E. coli* G6 expressing pMMB68, which encodes wild-type EtxB 23, were cultured at 37°C, induced with 0.5 mM isopropyl β-d-1-thiogalactopyranoside and lysed using a French Press (Thermo Electron Corporation, Waltham, MA, USA). After extensive testing of purification procedures, one method was adopted as optimal. Subsequently, EtxB (in 20 mM NaCl, 25 mM Tris–HCl, pH 8.0) was purified using cation and anion exchange chromatography (4°C, elution step gradient using 250 mM NaCl, 25 mM Tris–HCl, pH 8.0, with a 10 column volume 1,5-pentanediol wash during the first cation exchange), before lipopolysaccharide (LPS) depletion using Endotrap Red columns (Lonza, Walkersville, MD, USA). Purified EtxB contained ≤0.04 endotoxin units per μg protein as determined by a kinetic chromogenic *Limulus* amoebocyte lysate assay (AMS Laboratories, Silverwater, NSW, Australia). EtxB (1.58 mg/ml) was utilized either unheated or heat inactivated at 95°C for 10 min. in Eppendorf tubes and stored short-term at −20°C and long-term at −80°C in PBS.

### Generation of bone marrow chimaeras

To generate bone marrow chimaeras, 6-week-old C57BL/6J mice were lethally irradiated using two doses of 550 cGy, 3 hrs apart. Mice were rested for a few hours before being reconstituted *i.v*. with 5 × 10^6^ T cell-depleted CD11c-DTR-tg bone marrow cells. On the following day, mice were given 100 μl of anti-Thy-1 (T24) ascites intraperitoneally (*i.p*.) to deplete radioresistant T cells. Mice were given antibiotic water (10^6^U polymyxin B sulphate and 1.1 g neomycin sulphate; L sterile milliQ water) for 2 weeks following irradiation and left for at least 8 weeks before use.

### Preparation of cell suspensions

Bone marrow was flushed from the bone cavity using a 26G needle to deliver Dulbecco’s modified eagle medium supplemented with 10% FCS as described previously (sDMEM) 23. Spleen, lymph nodes (mediastinal and mesenteric) and bone marrow were dissociated by pressing tissue through a fine wire sieve. Red blood cell lysis, using a hypotonic buffer containing 140 mM ammonium chloride and 17 mM Tris-Base at pH 7.4 was performed on the spleen and bone marrow. For cell counting, trypan blue staining was used to exclude dead cells.

### Antibody staining

Cells were resuspended to a final concentration of 1–5 × 10^5^ cells/100 μl, plated in the wells of a 96-well microtitre plate, and pelleted by centrifugation. For FcR blocking, 25 μl FACS buffer containing 40 μg/ml of ‘Fc block’ specific for FcγII/IIIR (CD32/CD16; eBioscience, San Diego, CA, USA). Cells were washed twice with FACS buffer, resuspended in an antibody cocktail diluted in FACS buffer, incubated on ice for 20 min. and then washed in FACS buffer. Antibodies were all from eBioscience (San Diego, CA, USA), BD Pharmingen (San Diego, CA, USA) or BioLegend (San Gabriel, CA, USA), and included FITC- or APC-conjugated CD11c (clone N418), PE-Cy7-conjugated CD11b (clone M1/70), APC- PE- or FITC-conjugated CD8α (clone 53-6.7), PE- FITC- or biotin-conjugated I-A^b^ (clone 09/25/17), PE-conjugated CD80 (clone 16-10A1), biotin-conjugated CD86 (clone GL1), biotin-conjugated CD69 (clone 01502D), APC-conjugated CD172a (clone P84), APC-conjugated CD4 (clone GK1.5) and PE-conjugated TCR-Vα2 (clone B20.1). Where necessary, cells were incubated with a secondary conjugate on ice for 30 min. before washing and resuspension for flow cytometric analysis.

### Flow cytometry

Flow cytometry was performed on either a LSRII FACS machine (Becton Dickinson) or a FACS Calibur machine (Becton Dickinson, Franklin Lakes, NJ, USA). BD FACSDIVA software (Becton Dickinson) was used to set voltage parameters and event counts. For multicolour analysis, single colour controls were used to set compensation settings on the machine. FlowJo software (FlowJo, Ashland, Oregon, USA) was used to analyse data. Commonly, cell debris was gated out using a forward scatter (FSC) threshold of 100. Cells were further gated on the basis of side scatter (SSC) and absence of propidium iodide (PI) staining to detect live PI^−^ cells. Post-acquisition gating was used to obtain information on cell subsets. Staining with isotype control antibodies or ‘fluorescence minus one’ controls was used to set gates to distinguish specific antibody staining. Fluorescence-activated cell sorting was used to isolate splenic cell subsets based on marker expression. Spleen cells were prepared and stained with antibodies as described above. Sorting was performed on a BD FACSAria™ II (Becton Dickinson) cell sorter.

### Enrichment of T cells

Spleens and mesenteric lymph nodes (MLN) were obtained by sterile dissection from two mouse strains (C57BL/6.Tg(TcraTcrb)1100Mjb (OT-I) and C57BL/6.SJL/J.OT-II.CD45.1 (OT-II)) and placed in ice-cold modified Eagles’ medium containing 2.5% FCS (HEM2.5). Cell suspensions were prepared, washed and counted as above. Cells were resuspended in a cocktail of antibodies specific for Mac-3 (F4/80: rat IgG_2a_,κ; eBioscience), CD11b (M1/70: rat IgG_2b_,κ; eBioscience), Ter119 (TER-119: rat IgG_2b_,κ; eBioscience), Gr-1 (RB6-8C5: rat IgG_2b_,κ; eBioscience), major histocompatability complex class II (MHC-II; I-A/I-E; M5/114: rat IgG_2b_,κ; eBioscience), and either CD4 (GK1.5: rat IgG_2b_,κ; BD Pharmingen, BD Biosciences, North Ryde, NSW, Australia) or CD8α (53-6.7: rat IgG_2a_,κ; BD Pharmingen). Hereafter, CD8α will be referred to as CD8. Cells were incubated on ice before washing and incubation with pre-washed goat anti-rat BioMag magnetic beads (Qiagen, Clifton Hill, VIC, Australia). Tubes were incubated for 20 min. at 4°C with constant agitation. Additional HEM2.5 medium was added to cells and tubes placed on the magnet for at least 5 min. Unbound liquid was transferred to a fresh tube. Mouse tonicity PBS (MTPBS) was added as a column wash and cells resuspended in 10 ml MTPBS for storage on ice. To identify the purity of enriched CD4 or CD8 T cells, antibodies to Vα2 (B20.1: rat IgG_2a_,λ; BD Pharmingen) and either CD4 (GK1.5: rat IgG_2b_,κ) or CD8 (53-6.7: rat IgG_2a_,κ; BD Pharmingen) were allowed to bind to 100 μl aliquot of cells on ice for 30 min. before washing and analysis using flow cytometry. Typically, cell preparations were found to be 85–89% pure.

### Enrichment of dendritic cells by T/B cell depletion of spleen

Splenocyte preparations were made and counted as above. Cells were washed twice with 10-ml MACS buffer (PBS/5% BSA/2 mM EDTA) and then resuspended in MACS buffer (1 ml/10^8^ cells) for staining using antibodies to CD19 (eBio1D3; eBioscience), Thy1.2 (30-H12; eBioscience) and Ter119 (TER-119; eBioscience). Cells were incubated with antibody for 25 min. before washing twice with MACS buffer. Cells were resuspended in 1-ml MACS buffer containing MACS® anti-biotin microbeads (13 μl/10^8^ cells; Miltenyi Biotec, North Ryde, NSW, Australia) and incubated for 30 min. on ice. Cells were then washed, resuspended in 500 μl MACS buffer and added on to pre-washed MACS® LS columns, which were washed three times with 3-ml MACS buffer. Flow-through containing cells was collected, cells were washed and checked for depletion by antibody staining and flow cytometry.

### Enrichment of CD11c^+^ splenic DC

To isolate a population of CD11c^+^ splenic DC, splenocytes were prepared as above, red blood cells were lysed, and cells were counted and washed in MACS buffer. Cells were resuspended in MACS buffer (500 μl/10^8^ cells) containing anti-CD11c magnetic MACS® microbeads (10 μl/10^8^ cells; Miltenyi Biotec) with incubation on ice for 25 min. Cells were washed twice with 2 ml MACS buffer per 10^8^ cells. Before the second wash, cells were filtered through a 70 μm nylon sieve (Becton Dickinson). Collected cells were resuspended in 500 μl MACS buffer per 10^8^ cells, before loading onto a washed MACS® LS column. The column was washed as above to deplete unbound cells. The column was removed from the SuperMACS® magnet and labelled cells eluted with 5 ml MACS buffer using a column plunger to apply pressure.

### Culture of splenic DC

Dendritic cell populations were prepared by either T and B cell depletion of spleen or enrichment of CD11c^+^ DC in spleen or sorted to give pure DC subsets. Cells were either cultured at 2 × 10^6^ cells/ml in a total volume of 1 ml in 24-well flat bottomed plastic plates, or at 5 × 10^6^ cells/ml in a total volume of 200 μl in a 96-well plate with sDMEM. In some experiments, cultures were supplemented with EtxB wt (10 μg/ml), EtxB HI (10 μg/ml), LPS (3.3 μg/ml) or a combination of EtxB and LPS. Cells were then cultured for 12 hrs at 37°C in 5% CO_2_. The concentration of EtxB used *in vitro* was consistent with previously published studies 7,24.

### Generation of DC in Flt3 ligand-supplemented culture

Bone marrow cells were cultured at 2 × 10^6^ cells/ml in KDS RPMI medium in 6-well plates (Becton Dickinson) with addition of 200 ng/ml fms-related tyrosine kinase 3 ligand (Flt3L) derived as supernatant from transfected Chinese hamster ovary cells. Cells were cultured undisturbed in 10% CO_2_ at 37°C for 8 days. These cultures generate both cDC and pDC and these subsets can be delineated following antibody staining and cell subset identification using flow cytometry 25.

### T cell activation studies in CD11c-DTR-tg mice

For measurement of *in vivo* proliferation, cells isolated as described above were labelled with CFSE (Molecular Probes, Eugene, OR, USA) using 1 μl of CFSE (5-(and6-) carboxyfluorescein diacetate succinimidyl ester) stock solution (5 mM in DMSO) per 10^7^ cells. Vortexing was used to quickly and evenly distribute stain among cells, followed by incubation for 10 min. at 37°C. Labelling was terminated by the addition of 10 ml ice-cold HEM2.5 medium and cells were pelleted. Cells were washed twice with 10 ml ice-cold HEM2.5 before resuspension at 1 × 10^7^ cells/ml as CD8^+^ Vα2^+^ or CD4^+^ Vα2^+^ T cells taking into account per cent purity determined by flow cytometry. CD11c-DTR-tg mice harbour a gene that encodes the DTR gene receptor (DTR) as a green fluorescent protein (GFP) fusion protein under the control of the CD11c *Itgax* promoter. This model can be used to transiently deplete mice of CD11c^+^ cells by administration of small quantities of diphtheria toxin (Dtx) 26. To deplete CD11c^+^ cells, Dtx (Sigma-Aldrich) in PBS was administered *i.p*. at 4 ng/g bodyweight daily for 3 days, while controls received PBS. Mice were then vaccinated *i.n*. with 20 μg low-LPS OVA (Worthington Biochemical Corp, Lakewood, NJ, USA) freshly resuspended in PBS and half the cohort administered with 18 μg EtxB suspended in PBS consistent with doses used previously by others 27,28. At the same time, mice were given 2 × 10^6^ CFSE-stained OT-I or OT-II cells, isolated and stained as described above and injected *i.v*. in 200 μl into bone marrow chimaeras. Three days after vaccination and adoptive transfer of CFSE-labelled T cells, mice were killed and mediastinal lymph nodes and spleens harvested. Splenocytes were stained for CD11c to identify CD11c depletion level, and mediastinal lymph node cells stained for Vα2 and either CD4 or CD8, to identify adoptively transferred T cells. The per cent proliferating cells was determined from CFSE profiles within CD8^+^ Vα2^+^ or CD4^+^ Vα2^+^ populations. The absolute number of proliferated T cells was determined using the total lymph node cell count.

### Statistical analysis

Data are presented as mean ± SE for sample size *n*. Statistical significance was determined using one-way or two-way anova, or the unpaired two-tailed Student’s *t*-test as appropriate; *P* ≤ 0.05 was considered statistically significant. All analyses were performed with the Prism 5.0 program (GraphPad Prism, San Diego, CA, USA). The low yields of purified EtxB, as well as the scarcity of DC in tissues, limited the number of replicates which could be studied in any one experiment. For this reason, experiments were repeated two or three times to verify results.

## Results

### EtxB reduces the prevalence of myeloid and DC precursors in spleen

**Figure 1:**
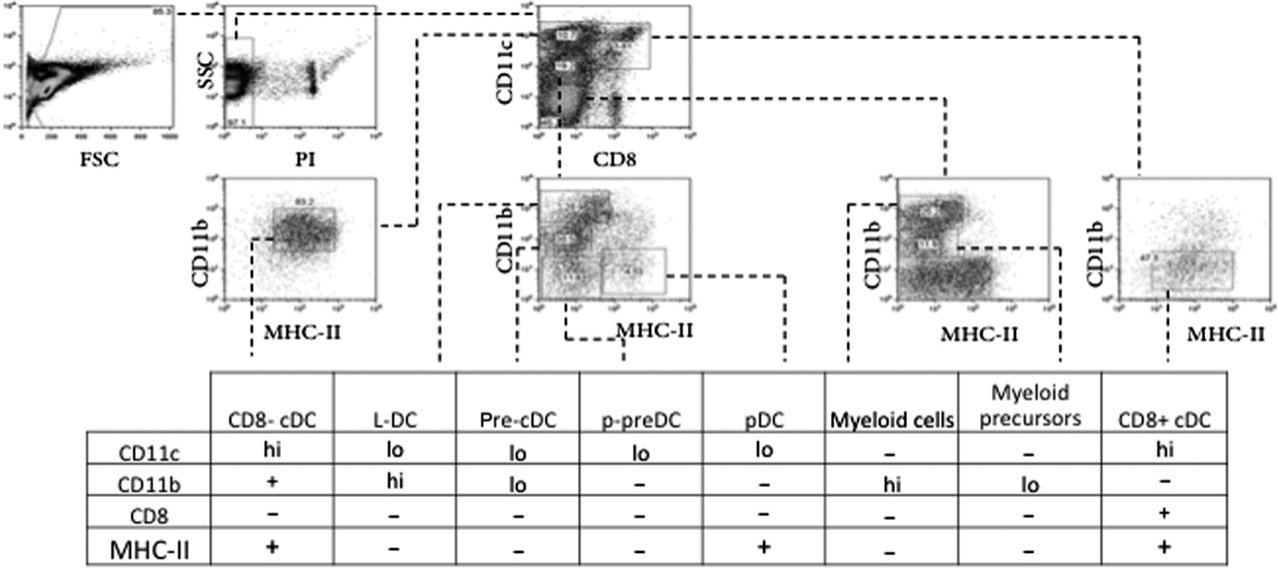
Identification of dendritic and myeloid subsets in spleen. Spleens were harvested from mice 24 hrs after receiving 18 μg EtxB, 18 μg heat inactivated EtxB (EtxB HI) or PBS as a control by *i.v*. inoculation in the tail vein. T and B cell-depleted splenocytes were stained with antibodies and propidium iodide (PI) for flow cytometric analysis. Plots from one representative experiment showing dendritic and myeloid subsets in spleen are shown. Cell debris was gated out using FSC *versus*SSC, dead cells were excluded by gating PI^−^ cells, and subsets identified using a combination of antibodies specific for CD11c, CD11b, CD8 and MHC-II. Gates were set on the basis of isotype control staining.

The effect of EtxB on dendritic and myeloid subset distribution *in vivo* was investigated following the exposure of mice to EtxB and investigation of changes in subset representation in spleen. C57BL/6J mice were exposed to potential activators *via* the tail vein, including EtxB, EtxB heat inactivated (HI) or PBS (control). Spleens were harvested at 24 hrs, depleted of T and B cells following lysis of red blood cells, and assessed for the presence of known cell subsets by flow cytometry following antibody staining. Common dendritic and myeloid subsets in spleen were identified on the basis of CD11c, CD11b, CD8 and MHC-II expression as shown in Figure1. These included CD8^−^ cDC (CD11c^hi^ CD11b^+^ CD8^−^ MHC-II^+^), CD8^+^ cDC (CD11c^hi^ CD11b^−^ CD8^+^ MHC-II^+^) and pDC (CD11c^lo^ CD11b^−^ CD8^−^ MHC-II^+^) subsets, gated as described in the literature 29,30, along with p-preDC 31. Myeloid cells were gated as the total population of CD11b^hi^ CD11c^−^ cells. L-DCs were gated based on their described phenotype as CD11c^lo^ CD11b^hi^ CD8^−^ MHC-II^−^ dendritic-like cells 32. Two further subsets were gated for the purposes of this study: DC precursors (CD11c^lo^ CD11b^lo^ CD8^−^ MHC-II^−^) and myeloid precursors (CD11b^lo^ CD11c^−^ CD8^−^ MHC-II^−^).

**Figure 2:**
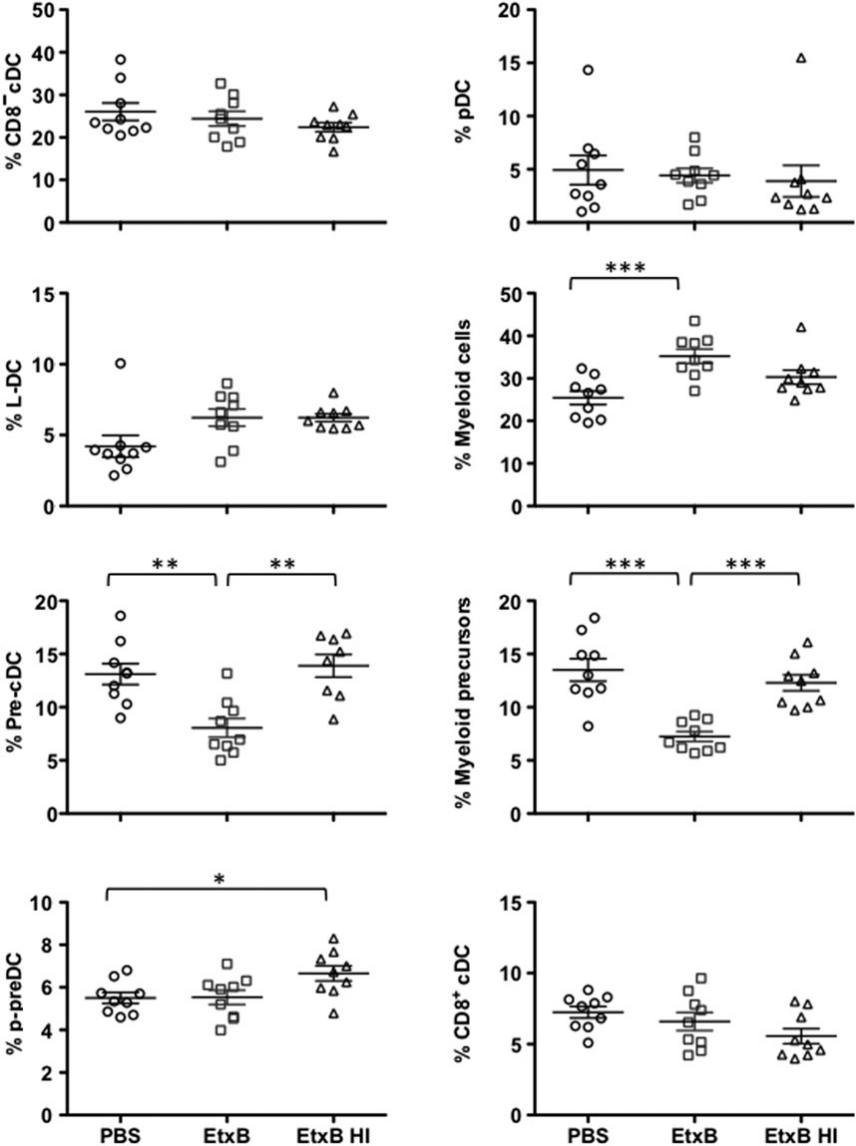
Representation of subsets in spleen. Mature dendritic and myeloid subsets, and dendritic and myeloid precursors, were gated as shown in Figure1. The proportion of each cell type is shown as a percentage of total dendritic and myeloid cells for nine individual mice. Means ± SE are shown by crosshairs. Significantly different pairs at *P* ≤ 0.05, *P* ≤ 0.01 and *P* ≤ 0.001 are identified by *, ** and *** respectively.

In response to 24-hr EtxB treatment, only the DC and myeloid precursor subsets in spleen were found to be significantly reduced (Fig.2). No changes were observed at 72 hrs post-EtxB (data not shown). This effect was EtxB-specific and was lost with EtxB heat inactivation. By comparison, no other DC subsets, including L-DC, pDC and cDC, were affected by treatment of mice with EtxB, although treatment with EtxB gave a significant increase in the number of myeloid cells in spleen. This was lost after heat treatment of EtxB and could reflect an inflammatory response. In resting animals, EtxB appears to accelerate the development of DC and myeloid cells from precursors, rather than induce a dramatic change in the representation of one or more particular subsets.

Mesenteric lymph nodes were also harvested from mice treated as above, and dendritic and myeloid subsets were identified to assess any changes in cell subset representation in comparison with spleen. CD8^−^ cDC, CD8^+^ cDC and pDC were identifiable based on CD11c, CD11b, CD8 and MHC-II expression, but no other CD11b^+^ or CD11c^+^ subsets were present. However, as the relative proportion of each subset did not change significantly at either 24- or 72-hr post-treatment, data have not been shown. The decrease in DC precursor numbers observed in spleen did not therefore relate to any change in DC prevalence in MLN.

### EtxB increases the number of immature DC

The effect of EtxB compared with LPS as an activator of mature DC was investigated by *in vitro* treatment. Spleen cells were prepared from mice by red blood cell lysis and T and B cell depletion. Cells were cultured in the presence of 10 μg/ml EtxB, 10 μg/ml EtxB HI, 10 ng/ml LPS, a combination of 10 μg/ml EtxB and 10 ng/ml LPS or the medium as a control (Nil). The concentration of EtxB and LPS used was informed by the literature and tested in trial experiments. An initial time course experiment over 24 hrs showed that 12 hrs was the time at which greatest change in cell viability and marker expression was detected after EtxB treatment (data not shown). All subsequent experiments therefore involved a 12-hr culture. Cells were stained for expression of CD11c, CD11b, CD8, MHC-II, CD80 and CD86 and analysed flow cytometrically. Subsets of CD8^−^ cDC and CD8^+^ cDC were gated on the basis of their CD11c^hi^ CD11b^+^ CD8^−^ MHC-II^+^ and CD11c^hi^ CD11b^−^ CD8^+^ MHC-II^+^ phenotypes as shown in Figure1. Expression of markers of activation in DC, including MHC-II, CD86 and CD80, was then determined by calculating median fluorescence intensity for MHC-II, and per cent positive cells for CD80 and CD86. Cell viability was also measured in terms of per cent cells excluding propidium iodide (PI^−^).

**Figure 3:**
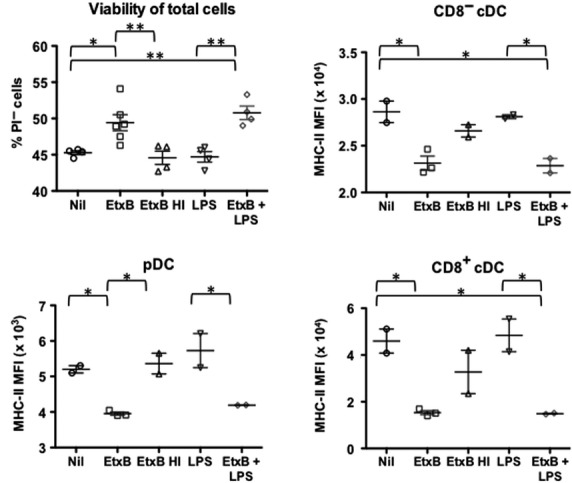
Effect of EtxB on DC survival and maturation *in vitro*. Spleens of mice were harvested, RBC were lysed and enriched for CD11c^+^ cells. Cells were cultured (5 × 10^6^ cells/ml) for 12 hrs with either 10 μg/ml EtxB, 10 ng/ml LPS, 10 μg/ml HI EtxB, a combination of EtxB and LPS, or medium as a control (nil). Cells were stained with antibodies to CD11c, CD11b, CD8, MHC-II, CD80 and CD86, or isotype control antibodies, prior to flow cytometric analysis. cDCs and pDCs were gated on the basis of CD11c, CD11b, CD8 and MHC-II expression as described in Figure1. Cell viability was measured by staining the total cell population with propidium iodide (PI). Expression of MHC-II is shown as median fluorescent intensity (MFI). Data are presented as mean ± SE of replicates shown as crosshairs. Results from this experiment are reflective of three similar experiments. Significantly different comparisons at *P* ≤ 0.05 and *P* ≤ 0.01 shown by * and ** respectively.

**Figure 4:**
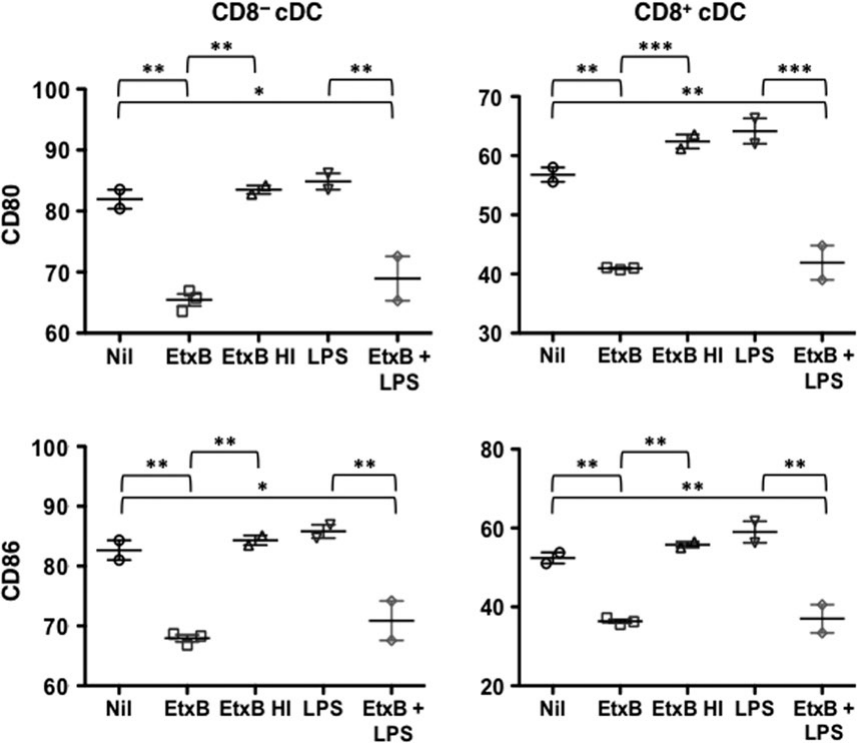
Effect of EtxB on DC activation status. Cells were prepared, cultured and analysed as described in Figure3. Expression of the CD80 and CD86 markers of DC activation was measured in terms of % cells showing antibody staining. Data are presented as mean ± SE of duplicates or triplicates shown as crosshairs. Results are representative of three similar experiments. Significantly different comparisons at *P* ≤ 0.05, *P* ≤ 0.01 and *P* ≤ 0.001 are shown by *, ** and *** respectively.

EtxB increased cell survival in *in vitro* culture, while LPS showed no specific effect on viability within 12 hrs (Fig.3). As cell survival was reduced following heat inactivation of EtxB, the effect seen must be specific for EtxB. Cultures of cells treated with EtxB or EtxB + LPS showed significantly lower expression of MHC-II compared with other cultures (Fig.3). Expression levels of the activation markers CD80 and CD86 were also reduced significantly after the culture of cells with EtxB, with and without LPS (Fig.4). These results could be interpreted to mean that EtxB acts to reduce the turnover and activation of mature DC resulting in higher numbers of immature DC. This could have an adjuvant effect by maintaining DC, as well as their antigen-presenting capacity for a longer period. The absence of any effect of LPS on DC present in spleen contrasts with the known effect of LPS in driving the development of DC from precursors or immature DC. However, this LPS effect is not evident within a 12-hr assay.

**Figure 5:**
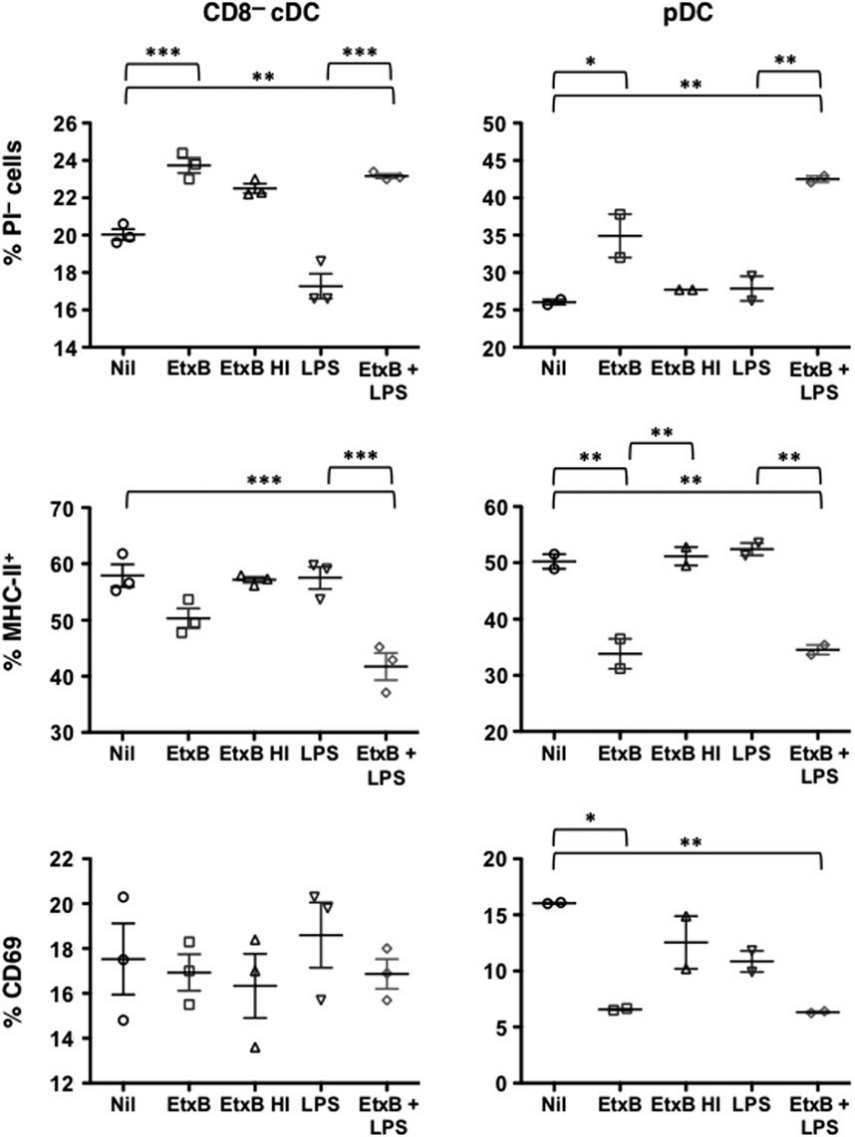
Direct effect of EtxB on sorted DC subsets in culture. Sorted subsets of CD11c^+^ CD11b^−^ CD8^−^ (pDC) and CD11c^hi^ CD11b^+^ CD8^−^ (CD8^−^ cDC) cells were cultured for 12 hrs with either 10 μg/ml EtxB or EtxB HI, 10 ng/ml LPS, a combination of EtxB and LPS, or medium as a control. Collected cells were then stained with antibodies to CD11c, CD11b, CD8, MHC-II and CD69 for flow cytometric analysis. Isotype control antibodies were used to set gates and to determine % positive cells. Propidium iodide (PI) staining was used to detect % live (PI^−^) cells. MHC-II expression was determined as MFI. Expression of CD69 is shown in terms of % cells staining. Data are presented as mean ± SE of duplicates or triplicates shown as crosshairs. Results are representative of two separate experiments. Significantly different pairs of data at *P* ≤ 0.05, *P* ≤ 0.01 and *P* ≤ 0.001 are represented by *, ** and *** respectively.

In further experiments, T and B cell-depleted splenocytes were sorted for isolation of pure populations of pDC and CD8^−^ cDC using marker expression as shown in Figure1. In general, CD8^+^ cDC were not readily recoverable in high enough numbers to be included in these experiments. Sorted pDC and CD8^−^ cDC were therefore cultured *in vitro* for 12 hrs with LPS and EtxB as described above. Cells were stained with PI to determine cell viability and with an antibody to detect MHC-II and CD69 expression by flow cytometry. Results obtained were consistent with those shown in Figures3 and 4 involving cultures of heterogeneous splenic myeloid cells. Increased cell viability was seen for both cell types cultured with EtxB, with no change following treatment with LPS (Fig.5). This was associated with reduced expression of markers of activation including MHC-II and CD69 for pDC, and of MHC-II for CD8^−^ cDC (Fig.5). Again, LPS had minimal effect on the expression of activation markers like MHC-II and CD69 over a 12 hr assay. This confirms the distinct effect of EtxB in supporting the viability of immature DC subsets as opposed to activated cDC and pDC.

### EtxB induces development of DC from precursors in Flt3L-supplemented BM cultures

*In vivo* studies showing a reduction in the numbers of DC and myeloid precursors following administration of EtxB (Fig.2) were interpreted to mean that EtxB acted to enhance the development of DC from precursors. A direct test of this hypothesis was therefore carried out. Flt3L-supplemented bone marrow cultures were established for 8 days. Under these conditions, DC precursors and immature DC have been shown to emerge 25. Cells were isolated after 8 days and cultured with either 10 μg/ml EtxB, 10 ng/ml LPS, EtxB + LPS or PBS as a control (Nil) for 24 hrs. Cells produced in culture were stained with antibody for identification of mature CD8^+^ cDC as CD11c^+^ SIRPα^−^ cells, and for CD11c^+^ SIRPα^+^ cells, representing the combined population of pDC and CD8^−^ cDC. The level of CD80, CD86 and MHC-II expression was then assessed on these subsets as a measure of cell development and activation.

**Figure 6:**
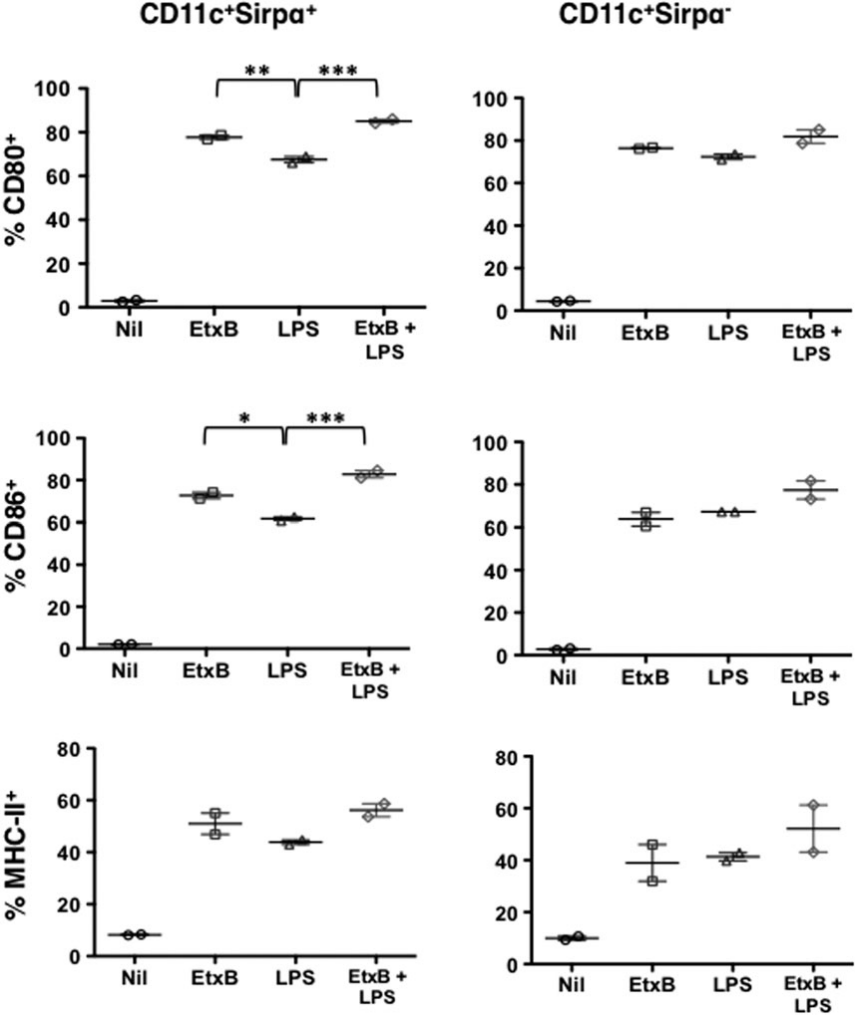
Effect of EtxB on development of DC in bone marrow cultures supplemented with Flt3L. Bone marrow cells were treated to lyse red blood cells and cultured (2 × 10^6^ cells/ml) in 200 ng/ml Flt3L for 8 days to induce DC development. The total cell population was then collected and incubated for 24 hrs with either EtxB (10 μg/ml), LPS (10 ng/ml), a combination of EtxB and LPS, or medium as a control. Cells were then collected and stained for CD11c and SIRPα to detect the CD11c^+^ SIRPα subset containing pDC and CD8^−^ cDC, and the CD11c^+^ SIRPα^−^ subset containing CD8^+^ cDC. Cells were stained for detection of the activation markers CD80, CD86 and MHC-II by flow cytometry. Isotype control antibodies were used to set gates. MHC-II expression was measured in terms of MFI, and expression of CD80 and CD86 as % cells staining. Data are shown as mean ± SE of duplicates represented as crosshairs. Significant differences between pairs at *P* ≤ 0.05, *P* ≤ 0.01 and *P* ≤ 0.001 are shown by *, ** and *** respectively.

Each of the three activation markers was found to be significantly up-regulated following the treatment of cells with EtxB, LPS or a combination of EtxB + LPS (Fig.6). CD80^−^ CD86^−^ MHC-II^lo^ cells reflecting precursor or immature DC predominated in untreated cultures of both CD11c^+^ SIRPα^−^ and CD11c^+^ SIRPα^+^ cells, suggesting that treatment with either LPS or with EtxB enhanced the development of mature DC from precursors showing higher levels of activation markers over a 24-hr assay. Small additional increases in CD80 and CD86 marker expression on CD11c^+^ SIRPα^+^ cells cultured with EtxB compared with LPS were also detected, with an additive effect because of the treatment with both. The increased maturation of DC shown here is consistent with evidence in Figure2 that DC precursor numbers are reduced in spleen following EtxB treatment of mice. Clearly EtxB acts on DC precursors, presumably inducing their development to give DC.

### Increased T cell activation since EtxB interacts with DC

**Figure 7:**
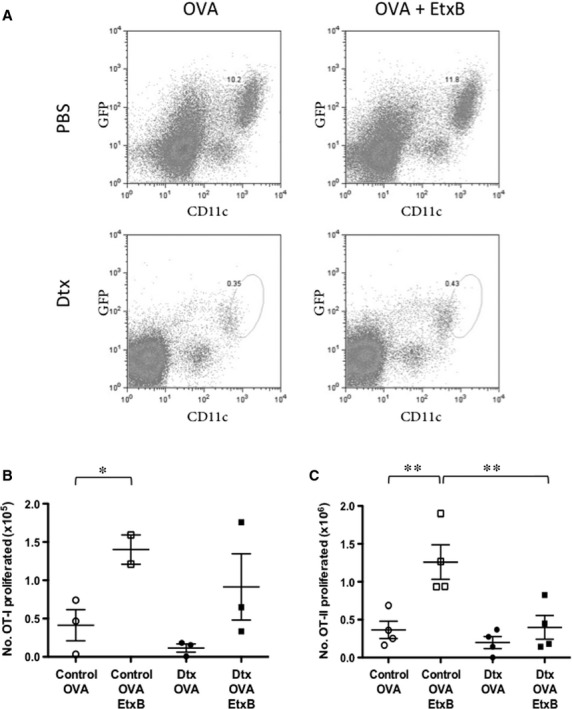
EtxB acts through DC to modulate T cell activation. Mice were lethally irradiated and reconstituted with 5 × 10^6^ T cell-depleted bone marrow cells from CD11c-DTR/GFP-tg mice. Chimaeras were then rested for 10 weeks. Diphtheria toxin (Dtx) was injected *i.p*. at 4 ng/g bodyweight daily for 3 days to deplete CD11c^+^ cells in chimaeras. Control mice received PBS. Mice were vaccinated *i.n*. on the first day with either OVA or OVA + EtxB. (A) Splenocytes from Dtx-treated and PBS-treated (control) CD11c-Dtx/GFP-tg chimaeric mice are stained for CD11c to confirm depletion of CD11c^+^ DC. Isotype control antibodies were used to set gates. (B) To assess T cell activation because of EtxB, 2 × 10^6^ CFSE-labelled CD8^+^ OT-I or CD4^+^ OT-II TCR-tg T cells were adoptively transferred *i.v*. into chimaeras. After 3 days, mediastinal lymph nodes were harvested and cells stained with propidium iodide (PI), and for TCR-Vα2, as well as CD8 or CD4, for flow cytometric analysis. Leukocytes were gated initially on the basis of forward and side scatter (FSC, SSC), then live (PI^−^) cells were gated to detect Vα2^+^ TCR-tg T cells for CFSE analysis. Number of proliferated T cells was calculated for groups of three or four mice. Number of proliferated OT-I or OT-II cells was determined by multiplying the total number of lymph node cells by the percentage of live, Vα2^+^ CD4^+^ OT-II or Vα2^+^ CD8^+^ OT-I T cells which had proliferated. Pairs of data significant at *P* ≤ 0.05 and *P* ≤ 0.01 are shown by * and **, with means and standard error of measurements shown as crosshairs.

The ability of EtxB to act as an adjuvant in T cell activation was then addressed. It has been suggested that EtxB has a direct immunomodulatory effect on DC as these cells are pivotal to antigen processing and presentation. An *in vivo* study was therefore undertaken using ‘CD11c depleting’ mice to determine the ability of EtxB to function as an adjuvant in antigen presentation. This would rule out an alternative hypothesis that EtxB interacts directly with T cells. In the CD11c-DTR/GFP-tg mouse, a single dose of 4 ng Dtx/g bodyweight delivered *i.p*. was sufficient to transiently deplete mice of CD11c^+^ cells within 24 hrs, with levels of CD11c^+^ DC recovering only after 3 days 26. As repeated application of Dtx can lead to death in CD11c-DTR/GFP-tg mice 27, haematopoietic chimaeras were generated by reconstituting lethally irradiated 6-week-old mice with T cell-depleted bone marrow from CD11c-DTR/GFP-tg mice 33,34. Chimaeric mice were then rested for 8–10 weeks after reconstitution before use in experiments. Figure7A shows CD11c depletion following three consecutive Dtx treatments. Gates transferred from plots of undepleted mice to Dtx-treated mice indicate change in CD11c *versus* GFP expression following DC depletion.

To assess the effect of EtxB on antigen presentation for CD4^+^ and CD8^+^ T cell activation, T cells from OT-II and OT-I TCR-tg specific for OVA_323-339_ and OVA_257-264_ (SIINFEKL) 35,36 were labelled with CFSE and adoptively transferred separately into chimaeric CD11c-DTR/GFP-tg mice pre-primed with OVA in the presence and absence of EtxB. Adoptively transferred T cells were identified in draining mediastinal lymph node by staining for CD4 or CD8, and Vα2. Both OT-I and OT-II cells express the Vα2 TCRα chain. The CFSE profile of Vα2^+^ CD8^+^/CD4^+^ cells was then used to assess T cell proliferation. These results showed that following OVA + EtxB administration to mice, CD8^+^ OT-I T cells exhibited greater levels of proliferation compared with OT-I T cells exposed to OVA alone. This effect was lost in two of three Dtx-treated animals depleted of CD11c (Fig.7B). A similar effect was observed for CD4^+^ OT-II T cells, with CD11c ablation almost completely negating the adjuvanting effects of EtxB (Fig.7B).

## Discussion

Previously, EtxB was shown to be a potent adjuvant for T cell activation, although the effect of this adjuvant on DC function was not known. Here, mice were treated with purified EtxB and the effect on DC subsets investigated *in vivo*. The relative prevalence of dendritic and myeloid subsets in spleen was found to be unaffected by EtxB treatment, with the exception of a decrease in percentages of DC and myeloid precursors. One interpretation of this result is that EtxB hastens the maturation of precursors in spleen, thus leaving reduced numbers of precursors. As the overall number of mature DC and myeloid cells in spleen or MLN remained constant, this also suggests that EtxB may increase the rate of turnover of these cells. This explanation does not preclude the possibility that newly formed mature DC have migrated into blood or other lymphoid organs following EtxB administration. Another explanation could be that, EtxB modulates precursors in bone marrow, slowing either their development, or their migration into blood and spleen.

The effects of EtxB on DC and myeloid subsets *in vivo* in resting animals are small and conservative, and are thought to be reflective of the effect of EtxB on DC development and function in the absence of an inflammatory stimulus. Previous studies involving inflammatory DC in a disease model (see e.g. ref. no 38) fall short of analysing the direct interaction between EtxB or cholera toxin (Ctx) and resting DC subsets as well as their immune function.

Current wisdom is that inflammatory agents like LPS activate DC leading to up-regulation of markers like CD80, CD86 and MHC-II which subsequently influence antigen-presenting ability. The effect of EtxB on the activation state of mature APC was therefore assessed by monitoring changes in the expression of activation markers following *in vitro* culture of freshly isolated CD11c^+^ splenocytes. For each of the mature DC subsets including pDC, CD8^+^ cDC and CD8^−^ cDC, EtxB treatment was found to significantly reduce expression of MHC-II, CD80 and CD86 at 12-hr post-treatment (Figs2 and 3). By 24 hrs after treatment, expression of these markers was equivalent in both treated and untreated cultures, suggesting that EtxB induces a transient change. This was not found for LPS which did not change the activation status of these mature DC subsets. While a recent report showed that LPS mediates down-regulation of CD11c on DC cultured *in vitro* 37, it was also found that EtxB did not alter CD11c or CD11b expression on splenic dendritic and myeloid cells *in vivo* (Fig.1). Similar results were observed when sorted pDC and CD8^−^ cDC were exposed to EtxB in culture, and likely reflects the ability of EtxB to delay or reduce culture-induced activation of cells (Fig.5).

The combination of increased cell viability and reduced activation could prolong the antigen presentation capacity of DC and consequently manifest as an adjuvant effect because of increased T cell activation. Indeed, this interpretation is not without precedent, as similar reports of EtxB increasing the viability and survival of B cells and macrophages for delayed antigen presentation have been reported 14,39. An alternative interpretation of the *in vitro* results could be that an increase in cell viability and a reduction in expression of activation markers is because of increased maturation of DC. This could also be reflected as increased development of DC from precursors following administration of EtxB *in vivo*.

All experiments exploring the effect of EtxB on DC activation and turnover also included LPS as a control, as it is a known and potent activator of DC. Importantly, experiments involving a combination of both EtxB and LPS showed a dominant effect of EtxB over LPS. This confirms that EtxB is acting through a different signalling pathway to LPS which is known to bind toll-like receptors on cells. These results also showed the more powerful effect of EtxB over LPS on mature splenic DC.

Heat inactivated EtxB also served as an effective negative control. Another suitable negative control could also be the G33D mutant of EtxB which no longer binds Gm1 ganglioside 2. This mutant is unable to induce EtxB-specific antibodies or influenza antigen-specific IgA responses compared with wild-type EtxB 2. However, attempts to purify both the H57A and the G33D mutant of EtxB which lack Gm1 ganglioside binding have been unsuccessful in this lab. Purification was not pursued because of low yields and high endotoxin contamination. Purification of mutants is, however, a future goal for assessment of the interaction of EtxB with mature DC and for investigation of EtxB immunomodulatory effects.

In contrast to mature DC *in vivo*, DC precursors isolated from Flt3L-supplemented bone marrow cultures exhibited increased maturation following EtxB treatment. The response of DC to EtxB treatment therefore appears to depend on the maturity of the APC. Again, the effect of EtxB on cells occurred independently of LPS. These different effects of EtxB on mature *versus* immature DC could also reflect differing levels of GM1 expression on the DC surface, a factor that warrants further investigation.

Finally, we also investigated whether EtxB-induced changes in the number of DC precursors, cell viability and activation marker expression on DC *in vivo* translated into changes in T cell activation. In an *in vivo* model for antigen delivery and presentation to OVA-specific TCR-tg T cells, EtxB did act as an adjuvant, showing significant OT-I and OT-II proliferation in normal mice over CD11c-depleted mice. The observation of EtxB-induced OT-I proliferation contrasts with previous reports of EtxB inducing apoptosis in CD8^+^ T cells 40, although those experiments involved *in vitro* exposure to EtxB. Comparison of responses in CD11c^−^-depleted *versus* normal mice confirmed the action of EtxB through direct interaction with cells. Those experiments highlighted the importance of CD11c^+^ DC in CD4^+^ T cell responses. This was less prominent for CD8^+^ T cells, but could be because of the presence of other MHC-I-bearing cells able to present antigen, particularly if the adoptively transferred T cells contained a population of memory T cells 41. In these experiments, only cellular proliferation was investigated. In other studies, EtxB was found to induce regulatory T cells 42, so further studies will investigate the phenotype and cytokine production of proliferating OT-I and OT-II cells to determine the type of T cell induced by EtxB-treated APC. One consideration is that CD11c is also expressed on other cell types including NK subsets with antigen-presenting capacity 43, and on some T cells, including regulatory CD8^+^ T cells 44. This should be taken into consideration in interpreting results in terms of a direct role for CD11c^+^ DC in mediating the EtxB effect.

The AB toxins are a class which includes Dtx, Ctx and heat-labile enterotoxin EtxB, under study here. Both Ctx and EtxB are similar in that their immunomodulatory capacity is mediated by the B subunit. Both have been shown to drive a Th1 inflammatory response to a Th2 response, making them targets for the control of autoimmune and inflammatory diseases 45. Most studies on the effect of toxins on DC have involved Ctx which enhances CD80 and CD86 costimulatory expression on murine DC 46. Cholera toxin also suppresses production of IL-12 by bone marrow-derived DC, which drives Th1 responses 47, and induces production of IL-6 and IL-10 important in Th2 differentiation. As shown here for EtxB, the adjuvant activity of CtxB is directly dependent on Gm1 ganglioside binding to cDC 48. Direct contact between cDC and Ctx was shown to be essential for adjuvant-mediated expansion of CD4^+^ T cells. Upon cDC depletion, CD4^+^ T cell expansion was found to be halted 48. While several studies have considered the effect of the closely related CtxB on DC, the effect of EtxB has not previously been evaluated in the literature. Here, EtxB has been shown to enhance the activation of both CD8^+^ and CD4^+^ T cells, consistent with its reported potent adjuvant activity 28. However, CtxB was found to only moderately enhance T cell responses 10, although chemical conjugation of CtxB to antigen did enhance the response further 49. Here, it has been found that DC are essential to the EtxB adjuvant effect.

One drawback with animal models for depletion of DC on the basis of CD11c expression is that CD11c is an imperfect marker. Multiple cell types including splenic metallophilic macrophages, marginal zone macrophages, alveolar macrophages, natural killer cells and plasmablasts are affected where the CD11c/*Itgax* promoter is used to deplete CD11c^+^ cells in mice 50–53. Here, the proliferation of T cells was assessed in the mediastinal lymph node following intranasal vaccination which precludes splenic marginal zone and metallophilic macrophage involvement, and alveolar macrophages found in pulmonary alveoli. It does not exclude plasmablasts or natural killer cells in the mediastinal lymph node. However, the CD11c/*Itgax* promoter-DTR-tg mouse strain represents a powerful tool to investigate the requirement for CD11c^+^ cells in *in vivo* immune responses.

In summary, EtxB is a potent adjuvant capable of activating T cells, which has the effect of inducing the development and maturation of DC rather than their activation, as well as increasing their viability. This could mean that EtxB treatment leads to more prolonged presentation of antigen to T cells, and greater T cell activation. EtxB also shows potential as a therapeutic agent, as it showed no toxicity towards DC, and only minor conservative changes in DC populations were evident.
